# Developing Farm-Level Post-vaccination Sero-Monitoring Systems for H5N1 Highly Pathogenic Avian Influenza in an Endemically Infected Country

**DOI:** 10.3389/fvets.2018.00324

**Published:** 2019-01-08

**Authors:** Peter A. Durr, Risa Indriani, Paul Selleck, Abdul R. M. Adjid, Tatty Syafriati, Jagoda Ignjatovic

**Affiliations:** ^1^CSIRO-Australian Animal Health Laboratory, Geelong, VIC, Australia; ^2^Indonesian Research Centre for Veterinary Sciences, Bogor, Indonesia; ^3^Faculty of Veterinary Science, University of Melbourne, Melbourne, VIC, Australia

**Keywords:** avian influenza virus subtype H5N1, avian influenza vaccines, haemagglutination inhibition test, highly pathogenic avian influenza, poultry vaccination

## Abstract

Whilst the serological responses of poultry following vaccination against highly pathogenic avian influenza H5N1 has been extensively investigated under laboratory conditions, there have been fewer studies conducted in the field. This applies particularly to the endemically infected countries routinely practicing vaccination, where the combination of multiple circulating clades and/or the use of vaccines with different seed strains makes the design and interpretation of field studies especially problematic. To address this for the particular situation of layer hens in the small to medium commercial sector in Indonesia, we developed a sampling regime before and after the vaccination given to point-of-lay pullets, and assessed serological response with a panel of test antigens. This confirmed that high titres were induced in those birds vaccinated with locally produced homologous H5N1 vaccines administered two or more times, but in flocks using imported heterologous H5N2 vaccines median titres were significantly lower, and unlikely to provide protection throughout the production cycle, without additional vaccination. Comparing the HI responses against the panel of antigens enabled the detection of the flock's exposure to different vaccine antigens, and made possible the detection of mislabelled vaccine seed strains. Furthermore, we show that test antigens need not be exactly matched to assess sero-protection in well vaccinated birds. Finally our study suggests that the POL vaccination serves as a useful reference point for following cohorts of layers throughout their production cycle, and thus enabling robust vaccination field effectiveness studies.

## Introduction

The epidemic of H5N1 highly pathogenic avian influenza (HPAI) virus in Indonesia was initially detected in poultry farms in central Java in August 2003 (1). Although the source of the virus has been traced by sequence comparisons back to Hunan province in southern China (2), the mechanism of the introduction has not been definitively determined. Initially it was suspected to be via migrating birds, but there is evidence that the pathway of introduction of the virus occurred through the transboundary movement of poultry and/or poultry products (3). Following this introduction, the disease spread rapidly, and by 2005 had been detected in poultry flocks in 30 out of Indonesia's 33 provinces (4).

Initially the Indonesian veterinary authorities attempted a stamping out policy, but when the extent of the spread of the disease became clear, this strategy was changed to one of vaccination (4). While vaccination proved successful in controlling the epidemic (5), there are a number of outstanding questions about its sustainability as a control measure (6, 7). Of particular concern is the extent to which antigenically variant strains are induced by vaccination, which in turn may lead to vaccine failures (8). In response to field reports that this might be occurring in Indonesia, Swayne et al. (9) undertook a challenge study using three Indonesian field strains and found that for one of these, *A/chicken/West Java/PWT-WIJ/2006*, all the tested vaccines were ineffective to prevent death in the challenged birds. This was supported by antigenic cartography which showed considerable drift from the then predominant strain used in the locally made vaccines, *A/chicken/Legok/2003*. Nevertheless, there are other reports of continued effectiveness of the vaccines, and this was supported by a field study in West Java demonstrating that vaccination could prevent disease in layer flocks and native chickens (10).

In a pilot survey of vaccination practices we conducted in 2008 in small to medium sized layer and broiler flocks in western Java, we confirmed that vaccination was being routinely used in the layer farms, but not in the majority of the broiler farms, which relied on biosecurity to prevent disease. With respect to the vaccination regimes used on the layer farms, these varied considerably, especially as regards the vaccine used and the number of vaccinations administered. Nevertheless, we did identify a consistent practice of giving pullets a HPAI vaccine at or around the point-of-lay (POL) stage, which was reported to occur between 16 and 20 weeks of age.

Arising from the initial survey a number of questions were posed, and accordingly we undertook a follow-up study with the general objective of providing baseline data to improve the advice on vaccination regimes. Specifically, in the layer flocks, we sought to establish the effectiveness of the POL vaccination to protect the birds during their early laying period. Due to the variety of vaccines being used, we recognized the need to undertake serological assays using antigens identical, or else closely matched to the vaccine strain. This required us to obtain detailed data about the farm management and vaccination practices, and to explicitly frame our study as an integration of field epidemiology and laboratory diagnostics, as well as extending and building on comparable studies being undertaken on vaccine efficacy and effectiveness at the same time within Indonesia (9–11).

## Materials and Methods

### Study Farms

**Figure 1:**
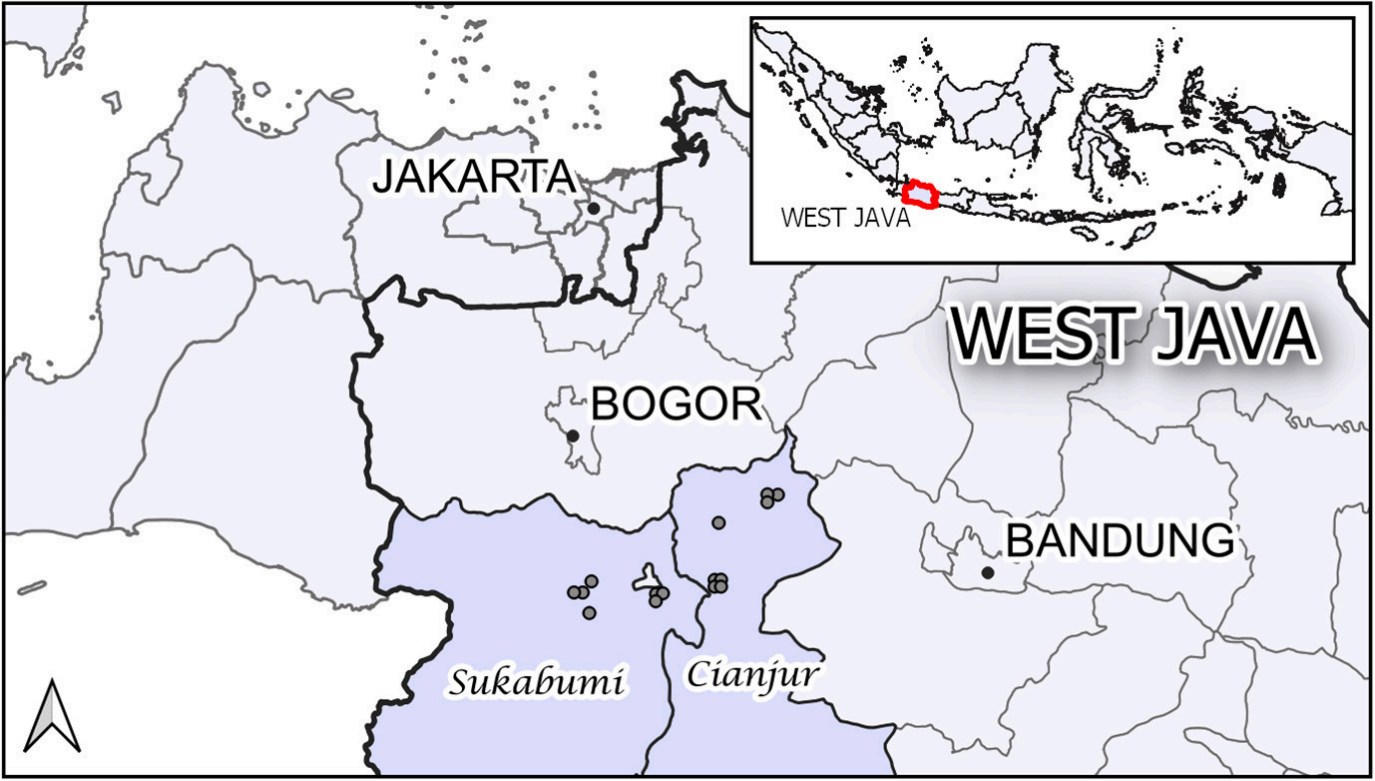
The location of the 15 study farms in the districts of Sukabumi and Cianjur in the province of West Java, Indonesia.

The study was carried out in 15 commercial layer poultry farms, all located in the districts of Sukabumi and Cianjur in the province of West Java, Indonesia (Figure 1). These districts have a well-developed poultry industry, being suitably placed to supply the large Jakarta market. Although some of the farms were large, all were considered by the district animal health office staff as Sector 3 under the FAO classification (12). The initial farm sampling visit occurred between December 2009 and January 2010.

The study farms were selected by officials of the local district animal health office (“Dinas Peternakan Kabupaten”), as the unavailability of a listing of commercial poultry farms in the two districts precluded random selection or formal sampling size calculations. However, there was no deliberate selection for any production or health criteria, although implicitly, participating farms tended to have good relations with the local animal health office. Thus, although not a random sample, the flocks were considered by the animal health staff to be representative of the layer farms in the area.

### Sampling

**Figure 2:**
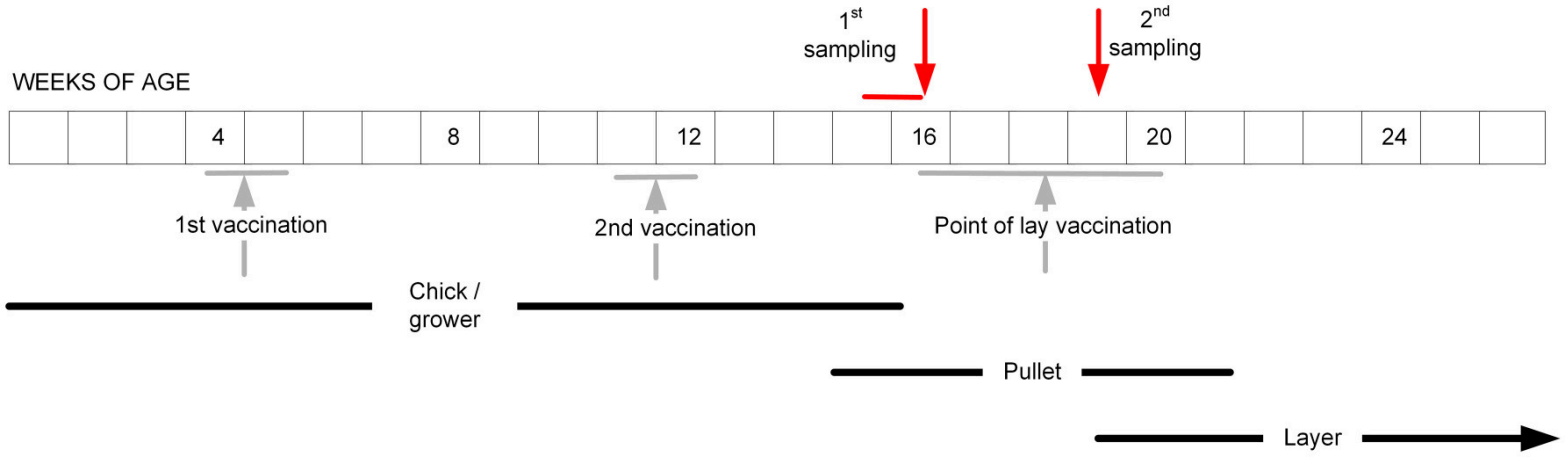
The intended sampling regime for the pullets in the study layer flocks, aiming to occur before and after the administration of the POL vaccine. In practice, the vaccination regimes on the farms turned out to be more variable than indicated (Table 1).

For the layer flocks, a structured sampling regime was developed to collect serum before and after the POL vaccination, which based on our pilot survey had been identified to be typically given when the pullets were between 16 and 20 weeks of age (Table 1). Farms were contacted to determine when this vaccination was intended to be administered for the next cohort of layers, and then visited ~1 week before this date (Figure 2). From each farm, 11 pullets were randomly selected, this sample size being chosen based on previous experience of estimating flock serological responses. From each bird, 0.5–1.0 ml of blood was collected from the wing (brachial) vein using a needle and syringe. Three to four weeks after vaccination, a second visit was undertaken and the procedure repeated. The birds were not individually marked, and therefore no attempt was made to resample exactly the same birds. However, the birds in the two samplings did belong to the same cohort.

**Table 1:** Summary of the farm survey questionnaire responses with respect to practices relevant to the study.

**Farm ID**	**Layer replacement (and age of purchase if growers/pullets)**	**Age at commencement of lay (weeks)**	**POL vaccine administered**	**Age vaccinations given (weeks)**
1	DOC	19	IND_2	4/17
2	DOC	20	CHI_3	5/15
3	DOC	19	MEX_1	6/17
4	DOC	20	CHI_2	4/9/12/17
5	DOC	19	IND_1	2/5/10/17
6	DOC	20	CHI_3	3/8/14/22
7	Growers (12w)	19	CHI_3	nk/14
8	Pullets (14w)	20	IND_2	4/12/16
9	DOC	19	MEX_1	6/17
10	Pullets (14w)	18	IND_1	nk/16
11	DOC and Pullets (14w)	19	CHI_1	DOC: 4/10/18 Pullets: nk/18
12	DOC	19	IND_2	3/9/18
13	DOC	20	CHI_2	4/9/20
14	DOC	19	CHI_3	4/9/20
15	Pullets (14w)	20	MEX_2	4/12/20

*DOC, day-old-chicks; nk, not known; POL, point-of-lay*.

At the time of the first blood sampling, a questionnaire was administered to the farm manager, to obtain data about the flock and the sampled cohort. This questionnaire had two broad sections: the first asking general details of the farm and the management of the pullets and layers, and the second about the HPAI vaccination practice, including the vaccine used for the POL vaccination (Table 1).

### Vaccines and Panel Antigens

For the 15 sampled farms, we identified seven vaccines being used for the POL vaccination (Table 2), and for each of these we determined the registered seed strain by reference to H5N1 vaccine listings (13, 14) or else by direct communications with the vaccine manufacturers. Antigens were then chosen to match these seed strains (Table 3), except for the IND_1 vaccine, for which we substituted a near identical isolate, *A/chicken/West Java/SMI-CSLK-EB/2006* (Table 4), as this was found to be more stable and thus provided a more consistent titer on culture. In addition to the four homologous antigens, we included in the panel one derived from an isolate representative of the then commonest circulating clade (“2.1.3.1”), *A/chicken/Konawe Selatan/BBVM204(O)/2007* (Table 3). Thus in the panel, one antigen was intended to be identical (“homologous”) to the seed strain, and the other four non-identical (“heterologous”).

**Table 2:** Anonymised details of the vaccines reported to be used by the farms for the sampled pullets, including the registered seed strain.

**Anonymised vaccine name**	**Country of production**	**Subtype/pathogenicity**	**Registered seed strain**	**Number of farms using**
CHI_1	China	H5N2–LPAI	A/turkey/England/N28/1973	1
CHI_2	China	H5N2–LPAI	A/turkey/England/N28/1973	2
CHI_3	China	H5N2–LPAI	A/turkey/England/N28/1973	4
IND_1	Indonesia	H5N1–HPAI	A/chicken/West Java/PWT-WIJ/2006	2
IND_2	Indonesia	H5N1–HPAI	A/chicken/Legok/2003	3
MEX_1	Mexico	H5N2–LPAI	A/chicken/Mexico/232/1994	2
MEX_2	Mexico	H5N2–LPAI	A/chicken/Mexico/232/1994	1

**Table 3:** Details of the antigens used for the HI test.

**HI Antigen**	**Isolate**	**Antigen abbreviation**	**H5N1 Clade**	**GenBank accession number for the HA gene**
Legok/03 H5N1	A/chicken/Legok/2003 (H5N1)	Leg/03	2.1.1	GU052426
CSLK-EB/06 H5N1	A/chicken/West Java/SMI-CSLK-EB/2006 (H5N1)	Cis/06	2.1.3.2	EU124276
Konawe/07 H5N1	A/chicken/Konawe Selatan/BBVM204(O)/2007 (H5N1)	Kon/07	2.1.3.1	Not deposited
England/73 H5N2	A/turkey/England/N28/1973 (H5N2)	Eng/73	n/a	EU636684
Mexico/94 H5N2	A/chicken/Mexico/232/1994 (H5N2)	Mex/94	n/a	AY497096

**Table 4:** Nucleotide percentage identity (green) and amino acid percentage similarity (red) distance matrices for the HA1 genes and proteins of the reported and presumed vaccine seed strains (Table 1) and the HI test antigens (Table 2).

	**Seed strain/HI antigen**						
	**Leg/03 (%)**	**Cis/06 (%)**	**Pwt/06 (%)**	**Kon/07 (%)**	**Eng/73 (%)**	**Mex/94 (%)**	**rgGD/96 (%)**
Leg/03		96.2	96.3	97.4	86.9	79.3	96.1
Cis/06	95.0		99.9	95.7	85.0	77.6	93.0
Pwt/06	95.0	100		95.8	84.9	77.5	92.9
Kon/07	98.4	96.0	96.0		85.6	79.1	93.7
Eng/73	95.7	91.3	91.3	94.7		82.2	89.8
Mex/94	92.9	88.8	88.8	92.2	94.4		80.6
rgGD/96	98.8	93.8	93.8	96.9	96.9	93.8	
**Heatmap classes**							
**Nucleotide identity**		**Amino acid similarity**					
>95%		>97.5%					
90–95%		95–97.5%					
<90%		<95%					

### Serology

All 330 collected blood samples were transported at room temperature to the Indonesian Research Center for Veterinary Sciences within 12 h. The serum was then extracted from the syringe, and transferred to a 1.8-ml Eppendorf tube, where it was then stored at −20°C. After preliminary testing to confirm the quality of the serum, the samples were then transported to the Australian Animal Health Laboratory (AAHL) where the Haemagglutination Inhibition (HI) tests were performed against the panel of selected antigens (Table 3). This testing used the standard AAHL SOP, which broadly follows that outlined in the OIE Terrestrial Manual of Diagnostic Tests (15). In brief, 25 μl of serum was diluted two-fold in PBS, starting from 1:4 and finishing at 1:2048, in U-bottomed microwell plastic plates and 4 HA units of antigen was added to each well. Following incubation at room temperature for 60 min, 50 μl 0.5% chicken RBC was then added to each well, and the plates were incubated at 4°C for 30–40 min to allow the RBCs to settle. Plates were read and the HI titer was determined as the value of the highest dilution of serum causing complete inhibition of the 4 HA units of virus.

### Data Analyses

For each individual HI result, negative titres (<1:4) were re-coded to have a value of “2,” and then each individual titer transformed to log_2_ titres for further analysis. The individual farm results of the sampled birds HI titres were highly variable, reflecting in part the fact that the sampling at the two visits were not from the same birds. Accordingly, for the purpose of our analysis we treated the HI titer results as a flock test, i.e., where the results are interpreted as estimating a flock-level parameter (16), by taking the median of log_2_ titres from the 11 sampled birds as the measure of the flock's serological status before and after the vaccination.

As anticipated, the vaccination caused increases in the median titer in the majority of the farms, but the actual extent of the increase was highly conditional on which test antigen was used. An added complexity was that some of the responses to these test antigens were highly correlated to each other. In order to provide a more complete analysis of the before and after responses, we treated the median responses for each farm to the test antigens as a multivariate response variable. The overall effect of the vaccination response was then tested for a significant increase using a multivariate analysis of variance (MANOVA). As a traditional sum-of-squares MANOVA is dependent upon the assumptions of multivariate normality, which was not shown to be met for our dataset, we conducted a nonparametric permutation MANOVA (“PERMANOVA”) to assure against Type I error (17). Aside for testing for an overall significant increase in median farm titres following vaccination, we also assessed the effect of the provenance of the vaccine on the increase in the titres. Following the detection of a significant provenance effect, we then used orthogonal contrasts to explore whether the Chinese-origin vaccines were significantly different in their responses to the Mexican-origin vaccines, despite both being registered as using H5N2 seed strains (Table 2). Finally, to explore the patterns of the HI titer profiles at an individual farm level, we undertook an unsupervised classification of the post-vaccination serological responses for each of the 15 farms, using a hierarchical (agglomerative) cluster analysis, with Ward's linkage used to assess inter-cluster dissimilarity.

All statistical analyses were done within the *R* statistical framework (v. 2.16 or v. 3.0), using either functions from the base or statistics libraries, or else from specialized packages. For the PERMANOVA we used the *Adonis* function of the *vegan* package (version 2.0–10), and for the cluster analysis we implemented the “*hclust*” function of the *cluster* package version 1.15.2.

#### Bioinformatics Analyses of HI Test Antigens

The clade classification of the H5N1 HI test antigens followed that outlined in the publication by the WHO/OIE/FAO H5N1 Evolution working Group (18). For each of these test antigens, as well as those subsequently presumed to be used as seed strains in the vaccines, a pairwise distance matrix determination was undertaken on alignments of the HA1 gene and its corresponding protein chain (Table 4). Nucleotide distances were determined by pairwise identity without assuming a substitution model, and amino acid similarity was calculated using the BLOSUM90 algorithm (19). Alignments and distance matrices were undertaken using *Geneious Pro* version 10.1.3 (www.geneious.com).

## Animal Welfare and Ethical Considerations

The sampling of the birds followed the standard practice used for the testing of flocks by the farm managers for their normal sero-monitoring and therefore was treated as a veterinary routine with benefit to the welfare of the birds, as a low titer would result in revaccination and therefore prevention of disease. The questioning of the farm managers was conducted in an ethical framework in which the following conditions applied: (i) the data collected was not of a personal nature; (ii) the data was anonymised before and during publication; (iii) the objectives of the survey were discussed with the farmers beforehand; (iv) taking part in the survey was voluntary and there was no implied pressure to participate; (v) there was no financial or social penalty for not taking part in the survey; (vi) the study was part of disease control research which benefits the farmers; and (vii) the results of the tests on the individual birds–and the wider survey results and their implications for post-vaccination monitoring–were communicated back to the farmers.

## Results

### General Properties of the Sampled Farms

All the farms were relatively large, with the median number of layers in production being 60,000 birds (range 30,000–138,000). Median egg production per month was 90,000 kg (range 27,000–204,000), with the median egg weight 64 g (range 60–65 g). The predominant breed was “ISA Brown” followed by “Lohmann Brown.”

Ten of the farms purchased only DOCs as their replacement strategy, while 4 purchased grower/pullets, and one farm purchased both DOCs and grower/pullets (Table 1). For those that purchased pullets, these mostly came from a single supplier, which was different for the five farms.

The median target age for pullets to begin laying was 19 weeks, and the median age for culling was 85 weeks. Premature culling of layers was low with a median of 2% (range 1–10%). Management practices were stable, with 14 of the managers reporting no change over the previous 3 years.

### Vaccination Practice for HPAI H5N1

With respect to HPAI H5N1, none of the farms reported outbreaks within the previous 3 years, indicating that the vaccination strategy they had adopted was effective. However, the vaccination regime used on the farms was more variable than indicated in the pilot study, both with respect to its timing and the vaccines used. The predominant practice (*n* = 9 farms) was to give the vaccination at the time of the start of laying, or up to 2 weeks beforehand (Table 1). Two farms gave the POL vaccination 1–2 weeks after the start of laying, and 4 farms gave the pre-laying final vaccination more than 4 weeks prior.

For the 11 farms that brought in DOCs (including the single farm that also brought in growers/pullets), 3 gave 4 vaccinations before or shortly after the POL, 4 gave 3 vaccinations and one 4 gave two vaccinations. For the 5 farms that brought in grower/pullets, the HPAI vaccination practice used by the supplier was known for two of these. These both administered a vaccine at 4 and 12 weeks to the growers, and were reported to be the same vaccine as was given by the farm at the POL.

Seven different vaccines were reported to be used by the farms (Table 2). These broadly fell within three groups: (1) Indonesian manufactured vaccines, using Clade 2.1 H5N1 seed-strains that had been isolated from outbreaks on farms in western Java; (2) imported Chinese manufactured vaccines registered as using a H5N2 LPAI seed-strain originally isolated from turkeys in England in 1973; and (3) imported Mexican manufactured vaccines using the *A/chicken/Mexico/232/1994* H5N2 seed-strain. All vaccines were oil emulsion, inactivated vaccines, sold as multi-dose bottles, and sourced from local suppliers. All vaccines were recommended by the manufacturer to be refrigerated when not in use.

All the farms reported to undertake post-vaccination sero-monitoring following the POL vaccination. The exact details regarding this were not asked in the questionnaire, but presumably followed standard practice of sending serum to a private laboratory to determine that the pullets had a titer greater than or equal to 1:16, the threshold which is generally taken to indicate sero-protection for poultry in Indonesia (4, 10, 20).

### Flock-Level Serological Responses Before and After POL Vaccination

**Figure 3:**
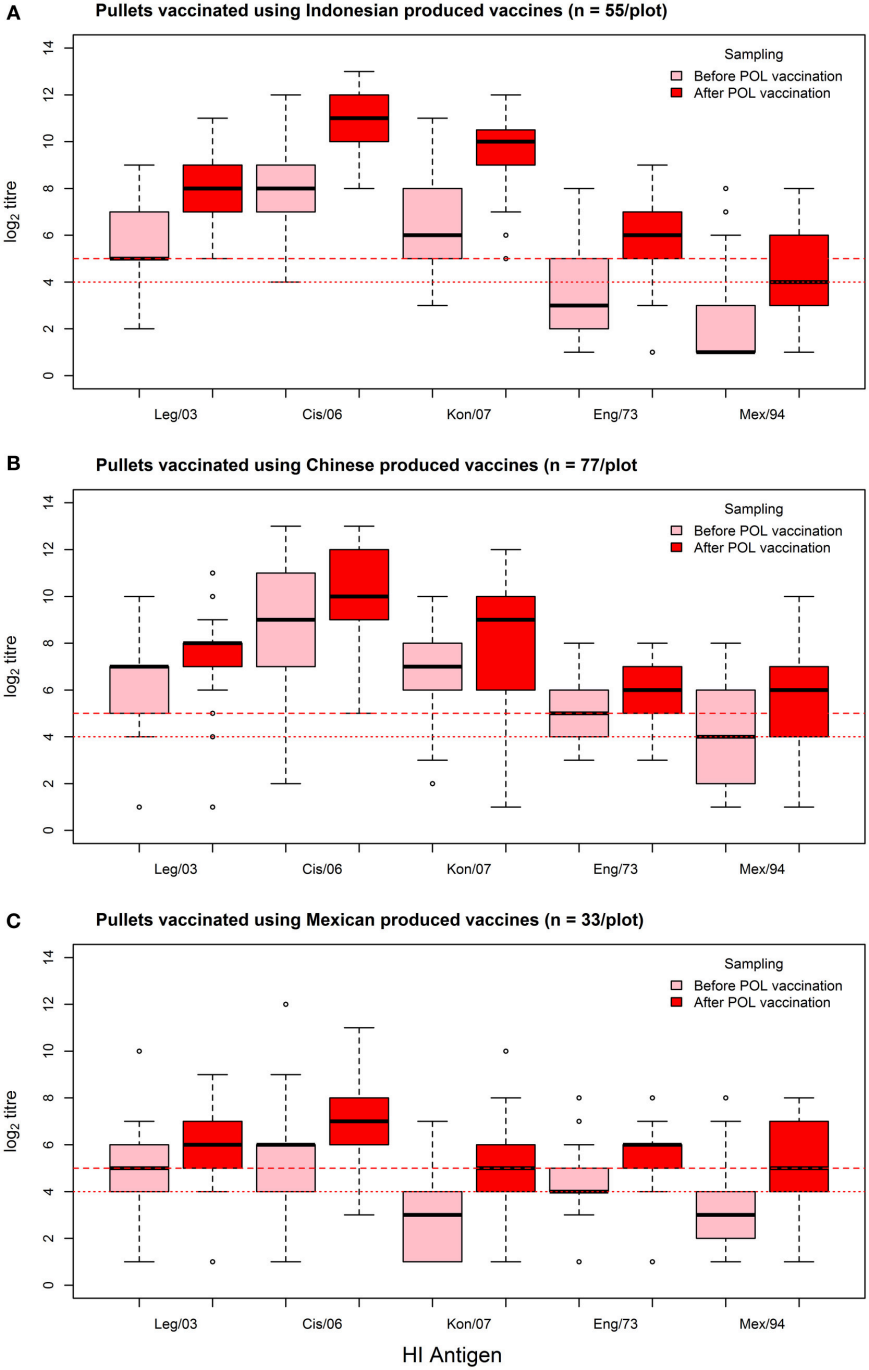
Box-plots of individual bird log_2_ titer responses against the panel of HI test antigens for the sampling before and after the POL vaccination, grouped according to the country of origin of the POL vaccine: **(A)** Indonesian vaccines, **(B)** Chinese vaccines, and **(C)** Mexican vaccines. Threshold titres for partial (1:2^4^) and full sero-protection (1:2^5^) are indicated with red dotted and red dashed lines respectively.

As assessed by the MANOVA, vaccination resulted in an overall increase in the flock median log_2_ titer (from 5.36 to 6.64, a 1.24 fold increase), which was highly significant (*p* < 0.01). This rise was most consistent for the Indonesian vaccines, wherein flock median log_2_ titer responses showed a 1.58-fold increase (from 5.22 to 8.27), irrespective of which test antigen was used (Figure 3, Table 5). The fold increase for the Chinese and Mexican vaccines was less than for the Indonesian vaccines, being 1.11 and 1.35 respectively. However, the Chinese vaccines had a much higher overall pre-vaccination baseline than the vaccines originating from Mexico (6.29 vs. 3.91), which was a highly significant difference (*p* < 0.01). There were however, considerable differences between the responses of the same serum when tested against the different antigens, with the H5N2 test antigens recording pre-POL vaccination titres below that of the international standard for sero-protection against mortality, *viz*. ≥1:32 (15).

**Table 5:** Median pre- and post-POL vaccination HI log_2_ titres for the 15 farms sampled in the study.

**Farm ID**	**POL vaccine probable seed strain**	**Pre-POL vaccination median HI titer**					**Post-POL vaccination median HI titer**				
		**Leg/03**	**Cis/06**	**Kon/07**	**Eng/73**	**Mex/94**	**Leg/03**	**Cis/06**	**Kon/07**	**Eng/73**	**Mex/94**
1	Leg/03 (H5N1)	**4.55**	6.45	5.36	2.09	1.09	**7.82**	10.36	9.27	5.00	3.82
2	rgGD/96 (H5N1)	5.18	6.73	5.55	3.55	1.82	8.27	10.45	9.00	5.82	5.36
3	Mex/94 (H5N2)	5.36	6.18	3.91	5.45	**4.27**	3.91	5.55	3.91	5.00	**3.91**
4	rgGD/96 (H5N1)	6.64	8.64	6.73	4.64	3.45	8.45	11.00	9.45	6.91	6.64
5	PWT-WIJ/06 (H5N1)	4.64	**6.64**	5.18	2.73	1.64	8.55	**11.27**	10.27	6.09	4.45
6	rgGD/96 (H5N1)	8.27	11.09	7.73	6.36	5.82	7.45	10.55	7.18	5.55	5.27
7	rgGD/96 (H5N1)	7.20	9.45	7.73	5.91	5.36	6.91	9.18	6.27	4.91	4.55
8	Leg/03 (H5N1)	**4.91**	7.64	6.27	2.09	1.09	**6.64**	10.45	9.09	4.73	3.64
9	Mex/94 (H5N2)	3.18	3.73	1.91	3.82	**2.18**	6.36	7.36	5.18	6.09	**5.45**
10	PWT-WIJ/06 (H5N1)	7.64	**10.45**	8.27	6.55	5.18	8.64	**11.64**	9.91	6.73	5.55
11	Eng/73 (H5N2)	5.36	6.27	4.73	**4.73**	3.27	5.82	6.45	4.73	**4.55**	3.27
12	Leg/03 (H5N1)	**6.73**	9.36	8.00	3.82	2.09	**8.27**	10.91	9.91	5.00	4.09
13	rgGD/96 (H5N1)	6.64	9.18	7.91	5.73	4.73	8.36	10.91	10.00	6.64	6.00
14	rgGD/96 (H5N1)	6.45	9.36	7.73	5.36	4.82	8.64	11.82	10.45	7.00	7.18
15	Mex/94 (H5N2)	5.09	5.91	2.55	4.00	**3.91**	6.45	8.18	5.55	5.55	**5.91**

*Homologous titres (i.e., where the HI test antigen titer matches the probable vaccine seed strain) are indicated by bold, highlighted text, except for the Chinese origin vaccines that were subsequently determined to contain a seed strain using A/goose/Guangdong/1/1996 (rgGD/96), as this conclusion was reached subsequent to the serological testing*.

**Figure 4:**
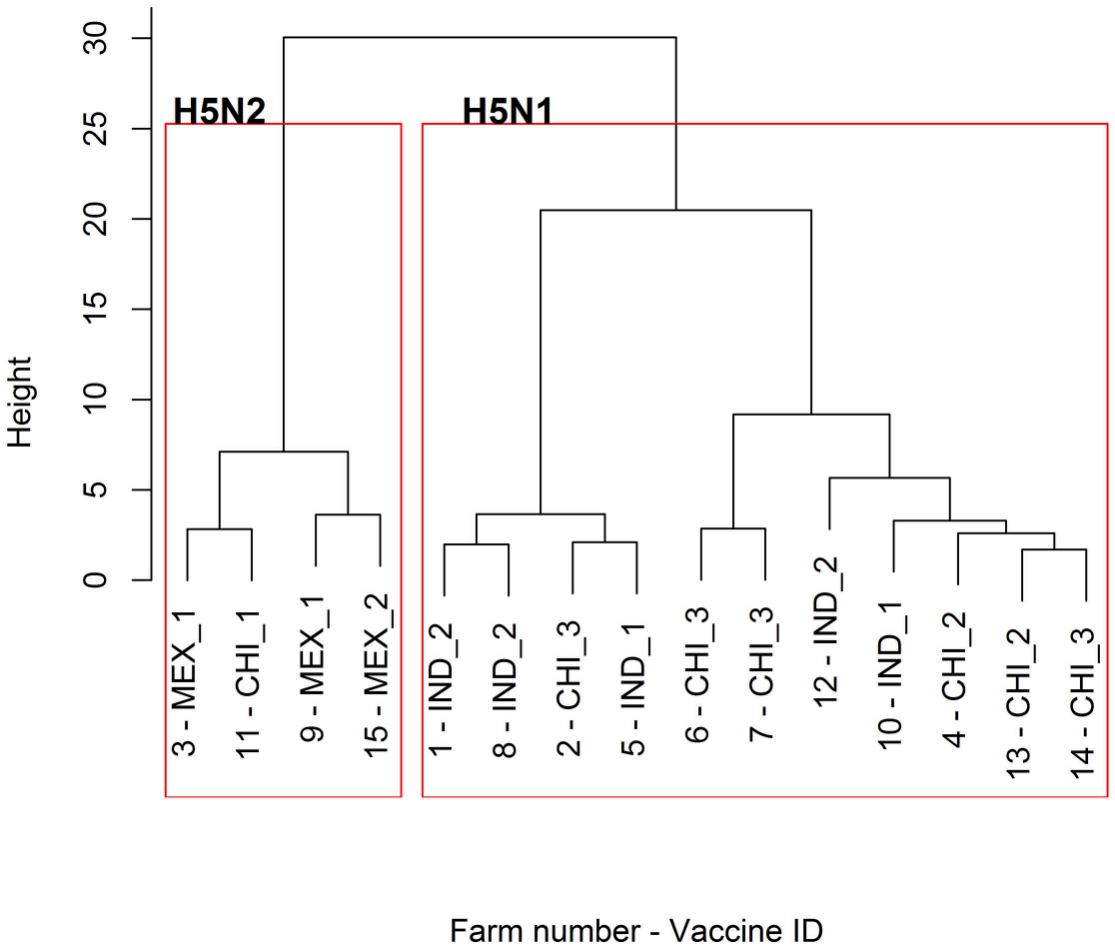
Dendrogram of the hierarchical cluster analysis of the HI profiles of the 15 sampled farms. For the provenance of the vaccines used for the POL vaccination on each of the farms, refer to Table 2.

The responses to the Chinese vaccines were highly variable (Figure 3), with responses intermediate between the Indonesian and the Mexican vaccines. The responses from the farm using the CHI_1 vaccine were similar to those of the farms using the Mexican vaccines, but the farms using the other two Chinese vaccines had responses comparable to the farms using the Indonesian vaccines, as was demonstrated with the hierarchical cluster analysis (Figure 4). This was consistent with the results of the orthogonal contrast analysis for provenance, which showed that the overall responses of the Indonesian-origin vaccines did not differ significantly from those originating from China, but these Chinese-origin vaccines were highly significantly different to the Mexican-origin vaccines (*p* < 0.01). Based on the evidence from the two different statistical analyses, it is concluded that two out of the three of the Chinese vaccines (CHI_2 and CHI_3) contained seed strains antigenically closer to H5N1 than the H5N2 for which they were registered, most probably a reverse genetics-generated H5N1 LPAI virus using the *A/goose/Guangdong/1/1996* isolate (rgGD/96) (9).

Adjusting the interpretation of the serological responses for the six farms which used the mislabelled Chinese-origin vaccines, it is possible to assess the capability of the vaccine seed strains to induce immunity in the early layer stage (i.e., after the POL vaccine) allowing for both the effect of the different test antigens and the two accepted thresholds for sero-protection (Tables 5, 6). This shows that all the 5 farms using the locally produced Indonesian vaccines had a median flock titer ≥1:32, which is the minimum recommended threshold to prevent mortality following exposure to HPAI viruses (15). Furthermore, this strong response to these vaccines were seen irrespective of which of the three H5N1 test antigens were used. Similarly there were strong responses to the vaccines surmised to contain rgGD/96, all producing median titres ≥1:32 when assessed using the H5N1 test antigens. However, for some of the Indonesian and Chinese H5N1 containing vaccines, the titres when assessed against the H5N2 test antigens were in the intermediate sero-protective range (i.e., ≥1:16, but <1:32), which undoubtedly reflects the antigenic distance between the subtypes (Table 4). Regarding the flocks which were vaccinated with H5N2 containing vaccines, one of these (Farm 3) had a titer below the sero-protective threshold when assessed against three of the test antigens, including the one to which it was homologous (Mex/94). This was not seen in the other flocks using the H5N2 vaccines, and may reflect other factors not accounted for in our analysis (due to insufficient replication) such as the timing and number of vaccinations (Table 1).

**Table 6:** Summary of median HI responses for the 15 sampled farms provided in Table 5 with respect to two thresholds for sero-protection (1:16 and 1:32) and three scenarios of matching between the test antigen and the probable seed strain used in the POL vaccine.

**Seed-strain vs. test antigen scenario**	**Sampling**	**Number of farms having a median HI titer above the two accepted thresholds for the HI test**	
		**≥1:16**	**≥1:32**
Single standard test antigen (Leg/03) (*n* = 15 farms)	Pre-POL vaccination	14/15	11/15
	Post-POL vaccination	14/15	14/15
Test antigen matched to the vaccine seed strain (*n* = 9 farms)	Pre-POL vaccination	7/9	3/9
	Post-POL vaccination	8/9	3/9
Test antigen corresponding to presumed circulating strain at time of study (Kon/07) (*n* = 15 farms)	Pre-POL vaccination	12/15	11/15
	Post-POL vaccination	14/15	13/15

Our use of a panel of antigens allows an assessment of the practice of the Indonesian testing laboratories of using a single standardized HI test antigen to assess the flock level of sero-protection, at both the lower (1:16) and upper (1:32) thresholds (Table 7). When the classification using standardized antigen (Leg/03) is compared with the commonest circulating strain (Kon/07), there was agreement for all flocks at the 1:16 threshold, and all but one for the 1:32 threshold, the latter being for flock 11, which used the CHI_1 vaccine containing a H5N2 antigen. Comparing the Leg/03 test antigen against the homologous test antigen, which would be expected to give the most accurate titer (21) is made complex by not having data for the presumed rgGD/96 containing vaccines, but for the 9 farms for which the homologous antigen data was available, the predictive value of the standardized antigen was similarly very high.

**Table 7:** Cross tabulation of results of the post-POL vaccination serology provided in Table 5 comparing the classification of the sampled flocks H5N1 HPAI sero-protective status using a standardized test antigen (Leg/03) against: **A**. one of the then commonest circulating strains (Kon/07); and **B**. the antigen homologous to the vaccine seed strain.

**A. Leg/03 vs. Kon/07 AT TWO THRESHOLDS OF POSITIVITY (*****n*** **=** **15 FARMS) AT TWO THRESHOLDS FOR POSITIVITY**							
**1. Threshold for positivity:** **≥1:16**		**Circulating strain (Kon/07)**		**2. Threshold for positivity:** **≥1:32**		**Circulating strain (Kon/07)**	
Leg/03		NEG	POS	Leg/03		NEG	POS
	NEG	1	0		NEG	1	0
	POS	0	14		POS	1	13
**B. A. Leg/03 vs. HOMOLOGOUS TEST ANTIGEN AT TWO THRESHOLDS OF POSITIVITY (***n*** = 9 FARMS**)							
**1. Threshold for positivity: ≥1:16**		**Homologous test antigen**		**2. Threshold for positivity: ≥1:32**		**Homologous test antigen**	
Leg/03		NEG	POS	Leg/03		NEG	POS
	NEG	1	0		NEG	1	0
	POS	0	8		POS	1	7

*Each cross-tabulation comparison uses the two thresholds applied to the HI test, i.e., ≥1:16 and ≥1:32*.

## Discussion

The HI test for the assessment of vaccine induced immune responses in poultry has a long history of usage, dating back over 50 years (22). By the time the H5N1 HPAI panzootic strain appeared in Hong Kong, it was a mature test, and thus used to assess the effectiveness of vaccination to prevent onward transmission of the disease (23). However, this use of the HI test describes a situation where the causative strain of the outbreak, the exact seed strain of the vaccine, the timing of the vaccination and prior exposure of the vaccinated birds are all known. A much more complex situation occurs when the disease is endemic, such as in Indonesia, where many of these variables might either not be known, or else subject to a degree of uncertainty. The challenge is to develop methods to assess the field performance of HPAI vaccination in the endemically infected countries, which at the time of our study, and to a degree to this day, remains an under-researched topic (24–27).

The approach we took was to assume that the vaccination system developed by the Indonesian poultry farmers, based on vaccinating the young birds several times before the commencement of laying, might be “fit for purpose,” and to make an objective assessment of its performance. For this, we developed a sampling regime centered on the POL vaccination, based on the assumption that this would be of most interest to the participating farms, and therefore achieve greater farmer cooperation. Furthermore, we developed an Indonesian language questionnaire survey to ensure the capture of quality data of the management and vaccination practices. Using this data we were then able to select antigens which corresponded to the registered seed strain of the POL vaccine, and then systematically compare responses to a panel of homologous and heterologous antigens.

In retrospect it can be seen that the limitation of this study design was the presumption that the seed strain used in the POL vaccine would equate with that to which it was registered, and because mislabelled vaccines were used in 6 of the 15 sampled farms, this made interpreting the resulting post-vaccination responses initially problematic. Through a combination of careful rechecking the farm questionnaire data, repeating the HI testing using newly obtained H5N2 antigens and undertaking detailed statistical analyses we arrived at the hypothesis that some of the Chinese origin vaccines contained a H5N1 antigen. This hypothesis was subsequently confirmed with the publication by Swayne et al. (9), reporting that some of Chinese origin vaccines being used in Indonesia at the time were found by sequencing to contain a seed strain using an antigen derived from *A/goose/Guangdong/1/1996* (H5N1).

The use of these mislabelled vaccines is now largely of historical interest, as in 2012 the Indonesian veterinary authorities prohibited the further use of imported vaccines. However, this example does illustrate the capability of the HI test, when used against a panel of homologous and heterologous antigens, to reconstruct the previous exposure of birds to vaccines. Although not unexpected, this is as far as we are aware the first time this result has actually been shown. A more practical learning from this vaccine mislabelling is that if inconsistent results are found in vaccine efficacy or effectiveness trials, then sequencing of the vaccine (and possibly the test antigen) is recommended.

Once the test results could be reinterpreted with the true or probable vaccine seed strain, then it was possible to confirm that those flocks in which a H5N2 vaccine was used had significantly lower median titres than those using a H5N1 seed strain. The use of H5N2 (and H5N9) vaccines had initially been recommended for use in Indonesia on the basis of the experience in Italy during an outbreak of H7N1 LPAI/HPAI between 1999 and 2001, where the application of a heterologous vaccine enabled the differentiation of vaccinated from infected flocks, and this assisted in proving freedom from disease in the latter stage of eradication (28). Our post-vaccination serological results are consistent with those obtained in the experimental challenge trials using H5N2 vaccines available in Indonesia (9) and thus provide additional evidence that the use of heterologous vaccines are suboptimal for the H5N1 endemically infected countries, and if a DIVA strategy is desired, then this would be best to be based on other testing methodologies (29, 30).

### Implications for Farm-Level Sero-Monitoring in Endemically Infected Countries

A learning from our field sampling that remains relevant to the Indonesian situation—and also to those endemically infected countries with multiple circulating HPAI viruses—is the appropriate selection of test antigens for the HI test in response to evolving H5N1 strains. The use of a panel of antigens in our study was based on the recommendation that only homologous antigens would provide a true estimate of the post-vaccination titer, and that heterologous antigens would give an underestimate (21). While obtaining accurate titres is important when vaccines are being assessed for both vaccine efficacy (31) and field effectiveness trials (32), they are of lesser direct interest when the purpose of the HI testing is simply to determine if the flock has an adequate post-vaccination level of sero-protection. This was clearly demonstrated in this study, where for the H5N1 vaccines, there was little difference in predictive value of a single standardized antigen as compared to the classification of the flocks using a homologous antigen and using an antigen matching the current circulating strain (Table 7). This has beneficial implications for the testing laboratories as using a single, standardized antigen is significantly more practical than varying the test antigen according to the vaccine used, as this avoids the complexity of keeping in stock various test antigens whilst assuring their quality over time. A similar argument applies to avoiding the need to change the test antigen to reflect the frequent identification of genetically variant H5N1 isolates, which have now been documented in all the endemic countries (18, 33–35). Nevertheless, there will be a need to carefully monitor the evolution of the predominant circulating virus and determine if it has diverged sufficiently to warrant a change. In the specific case of Indonesia, this is assisted by the creation of an avian influenza virus laboratory network, supported by a web-enabled database system, *IVM Online* (36), but for the endemic countries without comparable systems, approximate monitoring might be based on a bioinformatics analysis of the HA1 sequence (Table 4).

A second learning that has relevance beyond the Indonesian situation is that in layers the POL vaccination defines a reference point for the assessment of the effectiveness of HPAI vaccination throughout the birds' production cycle. Being able to define such a reference point is essential, because as was shown from our questionnaire survey (Table 1), the small to medium commercial layer sector engages in a diversity of practice, with respect to the vaccines used, the number administered and the timing of the vaccination. However, after the POL vaccination, the majority of the birds—with the exception of those being vaccinated with the H5N2 seed strain—had titres >1:32 and thus it becomes possible to make comparisons between flocks of vaccination parameters, such as the length of time the birds were protected, and the sero-protection status at the time of their culling. This possibility was in fact realized in a follow-on study, using some of the same farms reported on here, in which we were able to make a detailed assessment of the field effectiveness of HPAI vaccination by following cohorts of bird individually marked and resampled (32).

Finally, it needs to be stressed the limitation of our study with respect to understanding the effect of variables other than the seed strain in determining the HI titer in the pullets, *viz*. the type of vaccine, age at vaccination, number of vaccinations, the interval between vaccination and sampling etc. As was found, the farms used a diversity of vaccination practices (Table 1), and the relatively small sample size of our study precluded a detailed analysis of these. We do however, fully recommend that future studies explore the impact of vaccination practice on vaccine responses, both through more systematic field studies as well as complementary laboratory trials.

## Conclusion

Whilst the general principles for implementing sero-surveillance for the endemically infected countries relying on vaccination as the principal method of control are now established (7, 14, 27), it is also clear that each of these countries have unique challenges that require such sero-surveillance to be customized taking into account the specifics of the poultry production system, vaccination practices and permissible vaccines (5, 6, 37). This study assessing the use of the HI test system for POL pullets, and the follow-on one assessing sero-protection during and at the end of the layer production cycle (32), contribute to the evidence-base on which to provide recommendations for the commercial layer sector. It is however evident that developing robust sero-monitoring and sero-surveillance programs is a complex problem for which further research, both in Indonesia and beyond, is required.

## Author Contributions

PD: study design, data analysis, interpretation of results, writing of draft; RI: study design, farm visits, and specimen collection, data input; laboratory testing, interpretation of results; PS: laboratory testing, interpretation of results, editing of draft; AA: study design; TS: study design, farm visits, and specimen collection; data input; JI: study design, interpretation of results.

### Conflict of Interest Statement

The authors declare that the research was conducted in the absence of any commercial or financial relationships that could be construed as a potential conflict of interest.

## Abbreviations

DOC: day-old-chicks
FAO: Food and Agricultural Organization (of the United Nations)
HA: haemagglutination assay/haemagglutinin (gene)
HI: haemagglutination inhibition (assay/test)
HPAI: highly pathogenic avian influenza
LPAI: low pathogenic avian influenza
OFFLU: OIE/FAO influenza (network of expertise)
OIE: Office International des Epizooties (World Organization for Animal Health)
POL: point-of-lay
RBC: red-blood cells.
